# Enhanced Long-Term In-Sensing Memory in ZnO Nanoparticle-Based Optoelectronic Synaptic Devices Through Thermal Treatment

**DOI:** 10.3390/ma18061321

**Published:** 2025-03-17

**Authors:** Dabin Jeon, Seung Hun Lee, Sung-Nam Lee

**Affiliations:** 1 Department of IT Semiconductor Convergence Engineering, Tech University of Korea, Siheung 15073, Republic of Korea; 2 Department of Semiconductor Engineering, Tech University of Korea, Siheung 15073, Republic of Korea

**Keywords:** ZnO, nanoparticle, optoelectronic, synaptic device, annealing

## Abstract

Two-terminal optoelectronic synaptic devices based on ZnO nanoparticles (NPs) were fabricated to investigate the effects of thermal annealing control (200 °C–500 °C) in nitrogen and oxygen atmospheres on surface morphology, optical response, and synaptic functionality. Atomic force microscopy (AFM) analysis revealed improved grain growth and reduced surface roughness. At the same time, UV–visible spectroscopy and photoluminescence confirmed a blue shift in the absorption edge and enhanced near-band-edge emission, particularly in nitrogen-annealed devices due to increased oxygen vacancies. X-ray photoelectron spectroscopy (XPS) analysis of the O 1s spectra confirmed that oxygen vacancies were more pronounced in nitrogen-annealed devices than in oxygen-annealed ones at 500 °C. Optical resistive switching was observed, where 365 nm ultraviolet (UV) irradiation induced a transition from a high-resistance state (HRS) to a low-resistance state (LRS), attributed to electron–hole pair generation and oxygen desorption. The electrical reset process, achieved by applying −1.0 V to −5.0 V, restored the initial HRS, demonstrating stable switching behavior. Nitrogen-annealed devices with higher oxygen vacancies exhibited superior synaptic performance, including higher excitatory postsynaptic currents, stronger paired-pulse facilitation, and extended persistent photoconductivity (PPC) duration, enabling long-term memory retention. By systematically varying UV exposure time, intensity, pulse number, and frequency, ZnO NPs-based devices demonstrated the transition from short-term to long-term memory, mimicking biological synaptic behavior. Learning and forgetting simulations showed faster learning and slower decay in nitrogen-annealed devices, emphasizing their potential for next-generation neuromorphic computing and energy-efficient artificial synapses.

## 1. Introduction

Semiconductor memory is an essential component of modern automated devices, ranging from handheld electronics to supercomputers. These systems rely on temporary or permanent memory for data storage and processing [1,2]. In 2008, the Synapse project introduced memristors as synaptic devices with resistance-changing properties, paving the way for artificial synaptic networks [2]. Since 2016, research into optoelectronic synapses utilizing light has gained momentum, leveraging both inorganic and organic materials to enhance processing speed and reduce energy consumption compared to conventional electrically driven devices [3,4,5]. These advancements play a crucial role in neuromorphic computing and artificial intelligence by enabling energy-efficient, multi-signal processing capabilities [6,7]. Traditional electronic synaptic devices rely solely on electrical signals for information transmission, often suffering from high energy consumption, slow response times, and limited multi-signal processing capabilities [8,9,10]. In contrast, optoelectronic synapses leverage light stimulation, enabling ultrafast signal transmission, energy-efficient operation, and the ability to integrate both electrical and optical signals, making them highly advantageous for neuromorphic computing and artificial intelligence applications [11,12].

Various materials have been explored for optoelectronic synaptic devices, including perovskites, transition metal dichalcogenides (TMDs), and organic semiconductors. While perovskites exhibit strong light absorption and tunable bandgaps, they suffer from poor stability under ambient conditions [13]. TMDs, such as MoS_2_ and WS_2_, offer excellent flexibility and high carrier mobility but pose challenges in large-scale fabrication due to complex synthesis. Organic semiconductors provide flexibility and solution processability but often exhibit low carrier mobility and limited long-term stability [14,15]. Among these materials, ZnO stands out as a promising candidate for optoelectronic synaptic devices due to its wide bandgap (3.37 eV), high chemical stability, and excellent optoelectronic properties [16,17]. Its high transparency enables efficient UV absorption, while its strong exciton binding energy enhances photoresponse and resistive switching behavior [18]. Additionally, ZnO can be synthesized using low-cost, scalable techniques, making it suitable for large-scale neuromorphic applications. In particular, ZnO nanoparticles (NPs) have superior properties compared to bulk or thin-film ZnO due to their high surface-to-volume ratio, which enhances light absorption, charge carrier generation, and interfacial interactions [19]. The nanoscale dimensions of ZnO NPs provide a larger density of active sites for oxygen adsorption and desorption, crucial for modulating persistent photocurrent (PPC) and optoelectronic synaptic performance [20]. Additionally, ZnO NPs enable solution-processable fabrication, which is advantageous for flexible, wearable, and transparent neuromorphic devices. However, improving long-term memory (LTM) characteristics remains a key challenge for practical memory applications. Thermal annealing is widely employed to enhance crystallinity, reduce defects, and modify the surface structure, thereby improving charge transport and facilitating oxygen adsorption/desorption [18,21]. These modifications lead to higher carrier mobility and improved device performance. In this study, ZnO NP-based optoelectronic synaptic devices were fabricated and annealed under nitrogen and oxygen atmospheres at various temperatures. The optical and electrical characteristics were systematically analyzed, and learning processes were evaluated by varying UV exposure time, frequency, light intensity, and pulse number [22]. Additionally, optical resistive switching (RS) behavior, characterized by transitions between low-resistance and high-resistance states under UV exposure and electrical reset, was investigated. 

## 2. Materials and Methods

### 2.1. Fabrication of Metal/ZnO NPs Thin Film/Metal Optoelectronic Synaptic Devices

A 50 nm-thick ZnO NPs thin film was deposited onto a (0001) c-plane sapphire substrate using the spin-coating method at 2000 RPM for 60 s. The ZnO NPs ink (purchased from Sigma-Aldrich, Darmstadt, Germany), prepared with isopropyl alcohol as a solvent, was used. Subsequently, the ZnO NPs/ sapphire sample was thermally treated on a hot plate at 100 °C for 20 min to evaporate the solvent. To investigate the effect of different thermal treatment atmospheres and temperatures on the properties of the optoelectronic synaptic devices, the ZnO NPs thin films were annealed exclusively in nitrogen and oxygen atmospheres, without the use of Ar and H_2_, using a furnace set at 200 °C, 300 °C, 400 °C and 500 °C for 5 min each. After annealing, a 50 nm-thick Al electrode was deposited on each film using thermal evaporation at a deposition rate of 2.5 Å/s through a shadow mask. This process resulted in the fabrication of a metal- ZnO NPs thin film-metal structured optoelectronic synaptic device.

### 2.2. Characterization of ZnO NPs-Based Optoelectronic Synaptic Devices

Various analytical techniques were employed to analyze the surface, optical, and electrical properties of the ZnO NPs-based optoelectronic synaptic devices. Atomic force microscopy (AFM, NanoFocus my-Scope plus, Seoul, South Korea) was used to examine the surface characteristics of the ZnO NPs thin films, while ultraviolet (UV)-Visible spectroscopy (TermoFisher Scientific, Evolution 300, Waltham, MA, USA) and photoluminescence (PL) measurements, using laser excitation, were performed to assess their optical properties. The dark current and photocurrent of the ZnO NPs thin film-based optoelectronic synaptic devices were evaluated by measuring their response to a 365 nm UV light source. To investigate the resistive switching behavior during the optical set and electrical reset processes, UV light was applied under a bias voltage of 0.75 V. Additionally, the excitatory post-synaptic current (EPSC) of the optoelectronic synaptic devices was measured under a 3.0 V bias to assess various optical response conditions, including light exposure time, intensity, frequency, and number of exposures. Through these analyses, the comprehensive effects of thermal treatment temperature and atmosphere on the optical and electrical properties of the ZnO NPs thin films, as well as the overall performance of the optoelectronic synaptic devices, were systematically evaluated.

## 3. Results and Discussion 

### 3.1. Effect of Thermal Annealing Conditions on Surface Morphology, Optical Properties, and Defect States of ZnO NPs Thin Films

**Figure 1:**
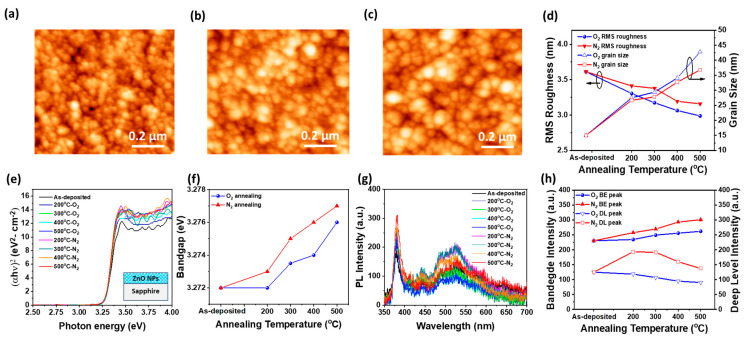
Surface morphologies of ZnO NP films measured by AFM for (**a**) as-deposited, (**b**) 500 °C-N_2_ annealed, and (**c**) 500 °C-O_2_ annealed samples. (**d**) RMS roughness of ZnO NP films as a function of annealing temperature and atmosphere. (**e**) Tauc plot (αhν)^2^ versus bandgap energy curves and (**f**) the extracted energy bandgap values of ZnO NPs films as a function of annealing temperature. (**g**) PL spectra, (**h**) bandedge (BE), and deep-level (DL) emission intensities of PL spectra for as-deposited, N_2_-annealed, and O_2_-annealed ZnO NP films as a function of annealing temperature ranging from 200 °C to 500 °C. The inset of (**e**) presents a schematic illustration of ZnO NPs on a sapphire substrate.

Figure 1a–c show the surface morphologies of as-deposited, 500 °C-N_2_ annealed, and 500 °C-O_2_ annealed ZnO NP films on c-plane sapphire (0001) substrates, respectively, as observed using AFM. The as-deposited ZnO NP film exhibits small grains with an average grain size of 15 nm and a root mean square (RMS) roughness of 3.61 nm, indicating a relatively uniform deposition by the spin coating method. As the annealing temperature increases from 200 °C to 500 °C, the surface morphology remains largely unchanged until 500 °C, where the surface grain size increases to 36.8 nm and 42.8 nm for 500 °C-N_2_ and O_2_ annealed films, respectively, as shown in Figure 1d. This grain growth results from enhanced atomic diffusion and recrystallization at elevated annealing temperatures, promoting the coalescence of smaller NPs into larger grains [23]. The effect is more pronounced in O_2_-annealed films due to oxygen incorporation, which stabilizes the crystal structure and further enhances grain growth. Meanwhile, the RMS roughness decreases slightly to 3.16 nm and 2.99 nm for ZnO NPs films annealed at 500 °C in N_2_ and O_2_, respectively. The reduction is attributed to improved crystallinity and densification during the annealing process, which smooths the surface by minimizing voids and irregularities. In particular, oxygen annealing facilitates defect passivation and surface energy minimization, resulting in a lower roughness and larger surface grains. Regardless of the annealing atmosphere, the increase in grain size and reduction in surface roughness with higher annealing temperature can be explained by Ostwald ripening [23]. This process involves the diffusion and mass transfer of material from high-energy interfaces to low-energy interfaces, driven by surface energy differences between smaller and larger ZnO NPs [24]. During high-temperature annealing, smaller ZnO NPs dissolve and migrate toward larger grains, leading to smoother surfaces. Oxygen-annealed films exhibit larger grain and lower roughness compared to nitrogen-annealed films due to oxygen diffusion into the ZnO crystal structure, which fills oxygen vacancies (V_o_), reduces defects, and decreases grain boundaries, thereby promoting the larger grain formation [25,26]. To analyze the optical properties of the ZnO NPs thin film annealed from 200 °C to 500 °C in nitrogen and oxygen atmospheres, absorption and PL measurements were conducted at room temperature, as shown in Figure 1e,g, respectively. Figure 1e shows the (αhν)^2^ versus photon energy curves of ZnO NPs thin film annealed at different temperatures and atmospheres, indicating that the photon absorption increases around 3.27 eV, regardless of the annealing temperature or atmosphere (nitrogen and oxygen). This value is very close to the typical bandgap energy of ZnO (3.3 eV). To evaluate the effect of annealing temperature and atmosphere on bandgap variation, the optical bandgap of the ZnO NP thin film was determined using the tangent extension method [16], where the bandgap was estimated by extrapolating the tangent line at the steepest part of the absorption edge to the x-axis. The bandgap of as-deposited ZnO NPs film is estimated to be 3.272 eV. However, the samples annealed in an oxygen atmosphere from 200 °C to 500 °C exhibited a gradual increase in the optical absorption coefficient and a slight bandgap shift from 3.272 eV to approximately 3.276 eV. In contrast, the ZnO NPs films annealed in a nitrogen atmosphere over the same temperature range showed a more pronounced increase in the optical absorption coefficient and a slightly larger bandgap shift compared to those annealed in oxygen, as shown in Figure 1f. This effect is attributed to the greater formation of oxygen vacancies in the nitrogen atmosphere, which increases the concentration of free electrons in ZnO. As a result, the bandgap expands slightly due to the Burstein-Moss effect [27,28]. In addition, this phenomenon is attributed to recrystallization occurring during the thermal annealing process, where high-temperature treatment in a nitrogen atmosphere promotes the formation of oxygen vacancies. This process facilitates defect redistribution and grain growth, leading to improved crystallinity and a more ordered crystal structure [22].

Figure 1g presents the room-temperature PL spectra of ZnO NP thin films annealed at different temperatures in nitrogen and oxygen atmospheres. The PL spectra exhibit band-edge emission around 3.3 eV, corresponding to the ZnO bandgap, along with defect-related emissions in the 2.8 eV to 2.0 eV range, attributed to crystal defects such as interstitial Zn and oxygen vacancies (V_o_) [29]. As shown in Figure 1h, the near-band edge (NBE) emission intensity was found to increase with an annealing temperature compared to the as-deposited film in both oxygen and nitrogen atmospheres. However, NBE emission was higher in nitrogen-annealed films than in oxygen-annealed films [30,31]. Additionally, deep-level emission gradually decreased with increasing annealing temperature in the oxygen atmosphere, which is attributed to the reduction in defects such as oxygen vacancies and interstitial Zn due to additional oxygen incorporation during the annealing process [32]. In contrast, deep-level emission initially increased at 200 °C–300 °C in the nitrogen-annealed films but decreased at higher temperatures. This increase at lower temperatures is likely due to defect formation associated with the formation of oxygen vacancies and Zn interstitials in the oxygen-deficient nitrogen atmosphere during low-temperature annealing. However, as the annealing temperature exceeds 400 °C, the overall enhancement of crystallinity outweighs the point defect formation, leading to a reduction in deep-level emission.

### 3.2. XPS Analysis of ZnO NPs Thin Films: Effect of Thermal Annealing on Chemical States and Defect Formation

**Figure 2:**
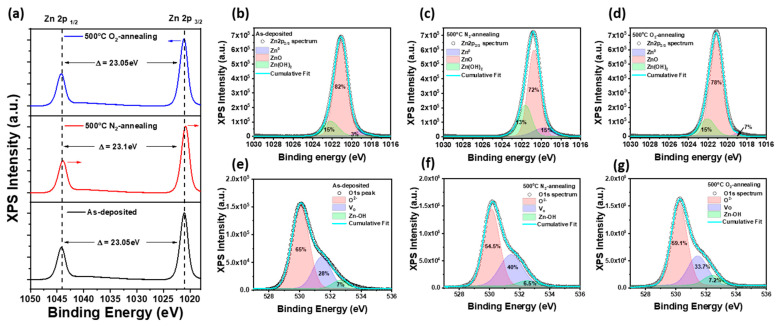
(**a**) XPS Zn 2p_1/2_ and 2p_3/2_ spectra of as-deposited, N_2_-annealed, and O_2_-annealed ZnO NPs. XPS Zn 2p_3/2_ spectra of (**b**) as-deposited ZnO NPs, (**c**) N_2_-annealed ZnO NPs, and (**d**) O_2_-annealed ZnO NPs, with fitting curves for Zn^2+^, ZnO, and Zn-OH components. XPS O 1s spectra of (**e**) as-deposited ZnO NPs, (**f**) N_2_-annealed ZnO NPs, and (**g**) O_2_-annealed ZnO NPs, with fitting curves for O^2−^, oxygen vacancies (V_o_), and Zn-OH components.

Figure 2a presents the XPS Zn 2p spectra for as-deposited, 500 °C-N_2_ annealed, and 500 °C-O_2_ annealed ZnO NPs thin films. The Zn 2p spectra exhibit two primary peaks corresponding to Zn 2p_3/2_ and Zn 2p_1/2_, which are characteristic of Zn^2+^ in the ZnO lattice [33]. The as-deposited ZnO NP film shows peaks centered at approximately 1021.8 eV (Zn 2p_3/2_) and 1045.0 eV (Zn 2p_1/2_), confirming the presence of ZnO without significant metallic Zn components. After annealing at 500 °C in both nitrogen and oxygen atmospheres, the Zn 2p binding energies remain relatively unchanged, indicating that the ZnO phase is maintained. The Zn 2p_3/2_ peak in the 500 °C-N_2_ annealed ZnO NP film shifts slightly toward lower binding energy (23.1 eV splitting) compared to the as-deposited sample, suggesting an increase in oxygen vacancies due to the reducing nitrogen atmosphere. The reduced coordination of Zn^2+^ ions with oxygen leads to a decrease in effective nuclear charge, lowering the binding energy. In contrast, the 500 °C-O_2_ annealed ZnO NP film exhibits a slight shift of the Zn 2p_3/2_ peak toward higher binding energy (23.05 eV splitting) compared to the nitrogen-annealed sample, closely resembling the as-deposited film. This shift indicates increased oxygen incorporation, as the oxygen-rich environment promotes oxygen adsorption and defect passivation. The higher electronegativity of oxygen enhances the effective positive charge on Zn atoms, resulting in a higher binding energy. In addition, as shown in Figure 2b,d the intensity of the peaks increases slightly for the oxygen-annealed ZnO NPs film, suggesting improved crystallinity and reduced defect density due to oxygen incorporation. In contrast, the nitrogen-annealed sample exhibits a slight peak shift and broadening, which may be attributed to an increased concentration of oxygen vacancies (V_o_) induced by the oxygen-deficient environment, as shown in Figure 2b,c. The O 1s spectra provide critical information regarding the oxygen bonding states within the ZnO lattice. As shown in Figure 2e–g, deconvolution of the O 1s peak reveals three distinct components: lattice oxygen (O^2−^) at ~530.2 eV, corresponding to oxygen atoms in the ZnO crystal lattice, oxygen vacancies (V_o_) at ~531.5 eV, associated with defect states due to oxygen deficiency, and chemisorbed oxygen species (Zn-OH) at ~532.6 eV, linked to surface hydroxyl groups or adsorbed oxygen [34]. The as-deposited ZnO film exhibits a relatively high fraction (~28%) of oxygen vacancies, as evidenced by the pronounced V_o_ component. A slight increase (33.7%) in V_o_ is observed in the oxygen-annealed ZnO NP films at 500 °C, suggesting that the oxygen-rich atmosphere limited the formation of oxygen vacancies, even under high-temperature annealing. Conversely, the nitrogen-annealed ZnO NPs film shows an increase in V_o_ intensity (40%), confirming that the nitrogen annealing process further promotes oxygen vacancy formation, which enhances carrier concentration and modifies the electronic properties of ZnO. These results demonstrate that thermal annealing in an oxygen atmosphere enhances ZnO crystallinity by reducing defect states, while nitrogen annealing increases oxygen vacancies, potentially influencing electrical and optoelectronic performance.

### 3.3. Effect of Thermal Annealing on Dark and UV Photoresponse in Al/ZnO NPs/Al Optoelectronic Synaptic Devices

**Figure 3:**
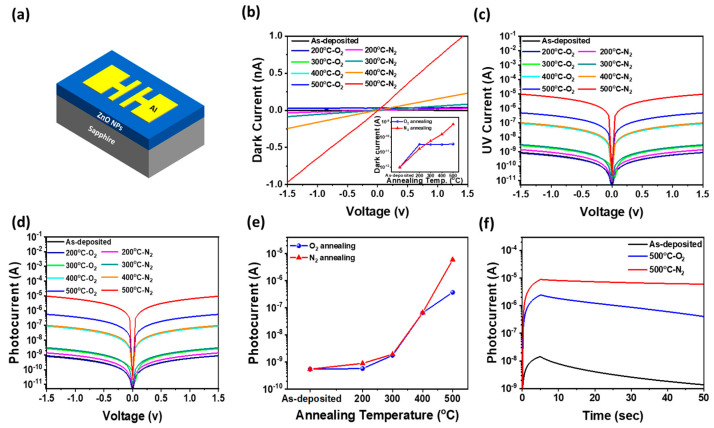
(**a**) Schematic diagram of an Al/ZnO NPs/Al optoelectronic synaptic device fabricated on a sapphire substrate. (**b**) Dark current, (**c**) UV current, and (**d**) photocurrent of the two-terminal device annealed at different temperatures (200 °C–500 °C) in O_2_ and N_2_ atmospheres. (**e**) Photocurrent values of Al/ZnO NP/Al devices with different annealing atmospheres as a function of annealing temperature ranging from 200 °C to 500 °C. (**f**) Logarithmically scaled photocurrent as a function of time after 5 s of UV exposure followed by UV off for Al/ZnO NPs/Al devices annealed under different conditions.

Figure 3a presents a schematic diagram of an Al/ZnO NPs/Al optoelectronic synaptic device fabricated on a sapphire substrate. Figure 3b shows the current (I)-voltage (V) curves measured in the dark for the Al/ZnO NPs/Al optoelectronic synaptic device, where ZnO NP thin films were annealed at temperatures ranging from 200 °C to 500 °C in nitrogen and oxygen atmospheres. The as-grown Al/ZnO NPs/Al devices, without the annealing process, exhibited an extremely low dark current of approximately 1.0 pA at 1.0 V. In contrast, devices annealed in an oxygen atmosphere at temperatures ranging from 200 °C to 500 °C showed only a slight increase in dark current with rising annealing temperature, remaining within a few tens of pA. However, Al/ZnO NPs/Al devices annealed in a nitrogen atmosphere exhibited a significant increase in dark current, rising from 1.0 pA to 709 pA at 500 °C as the annealing temperature increased, as shown in the inset of Figure 3b. This substantial increase in dark current under nitrogen annealing, compared to oxygen annealing, can be attributed to several factors. In an oxygen atmosphere, the annealing process reduces the concentration of oxygen vacancies within ZnO NPs, as oxygen molecules diffuse into the lattice and fill these vacancies, as shown in Figure 2e–g. This passivation of defects suppresses free carrier generation, limiting the increase in the dark current [19]. Conversely, annealing in a nitrogen atmosphere results in a substantial increase in dark current as the annealing temperature increases. This effect is primarily attributed to the accelerated formation of surface point defects, which facilitate the grain growth in ZnO NPs through diffusion and recrystallization processes [20]. The increase in grain size, along with the associated surface defects, enhances carrier concentration, thereby elevating the dark current. Figure 3c presents the UV current-voltage characteristics of the Al/ZnO NP/Al device under exposure to a 365 nm UV light source. The UV current at 1.0 V for the as-grown Al/ZnO NP/Al optoelectronic synaptic device was very low, measuring only 0.55 nA. However, it increased with rising annealing temperatures in both nitrogen and oxygen atmospheres, attributed to the improvement in optical properties due to the reduction in crystal defects in ZnO NPs. Specifically, in an oxygen atmosphere, the UV current increased from 0.58 nA at 200 °C to 322 nA at 500 °C, demonstrating enhanced photoresponse with higher annealing temperature. In contrast, in a nitrogen atmosphere, the UV current showed a significantly greater increase, rising from 0.91 nA at 200 °C to 6.01 μA at 500 °C. Furthermore, the photocurrent, defined as the difference between the UV current and the dark current, increased with rising annealing temperature regardless of the annealing atmosphere, as shown in Figure 3d. However, the increase in photocurrent was much more pronounced in the nitrogen-annealed samples compared to oxygen-annealed ones, as shown in Figure 3e. The dark current remained within the range of a few pA to tens of pA, which is relatively negligible compared to the UV current, regardless of the annealing temperature or atmosphere. As a result, the photocurrent was nearly equivalent to the UV current. The significantly higher UV current and photocurrent observed in nitrogen-annealed samples across all temperatures are attributed to the increased formation of oxygen vacancies and surface defects during nitrogen annealing. These defects act as shallow donors, increasing the carrier concentration and enhancing light absorption and photogenerated carrier dynamics. Additionally, the nitrogen annealing process promotes grain growth and improves crystallinity, further contributing to enhanced UV response. 

Figure 3f presents the logarithmically scaled photocurrent as a function of time after 5 s of UV exposure followed by UV off for Al/ZnO NP/Al devices under different annealing conditions. The as-deposited devices exhibited very low photocurrent levels and rapid decay after UV was turned off. However, with increasing the annealing temperature to 500 °C, the photocurrent significantly increased to the μA range, and its decay remained slow even after UV shutdown. Notably, the device annealed in a nitrogen atmosphere exhibited higher photocurrent generation and a slower decay compared to the oxygen-annealed device. This behavior confirms that ZnO NPs annealed in nitrogen not only developed deep-level-related grain boundaries, where photoexcited carriers were effectively trapped, but also exhibited increased oxygen vacancies, as confirmed by XPS analysis. These oxygen vacancies introduce deep-level defect states within the bandgap, which serve as trapping centers for photogenerated carriers, thereby enhancing persistent photoconductivity (PPC). Additionally, nitrogen annealing resulted in reduced surface oxygen adsorption, minimizing photocurrent suppression caused by oxygen readsorption and sustaining the photocurrent for an extended period—a characteristic known as PPC. These results suggest that ZnO NP devices annealed in a nitrogen atmosphere can retain photoexcited carriers for an extended duration, indicating their potential for long-term memory applications in optoelectronic synaptic devices.

### 3.4. Resistive Switching Behavior of Al/ZnO NPs/Al Optoelectronic Synaptic Devices: Influence of Thermal Annealing on Optical Set and Electrical Reset Processes

**Figure 4:**
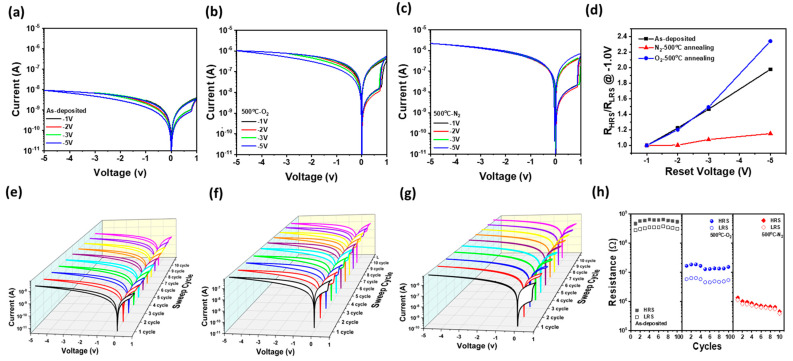
Optical resistive switching I-V curves of (**a**) as-deposited, (**b**) 500 °C-O_2_ annealed, and (**c**) 500 °C-N_2_ annealed Al/ZnO NPs/Al devices under various electrical reset conditions (Reset voltage from −1.0 V to −5.0 V). (**d**) The ratio of high-resistance state to low-resistance state (R_HRS_/R_LRS_) at −1 V, obtained from resistive switching curves with increasing electrical reset voltage from −1 V to −5 V for Al/ZnO NPs/Al devices with different annealing conditions. Optical resistive switching I-V curves of (**e**) as-deposited, (**f**) 500 °C-O_2_ annealed, and (**g**) 500 °C-N_2_ annealed Al/ZnO NPs/Al devices after 10 switching cycles. (**h**) Variation of HRS and LRS values at −1.0 V in repeated RS I-V measurements for as-deposited, 500 °C-O_2_ annealed, and 500°C-N_2_ annealed devices.

Figure 4a–c show the resistive switching (RS) behaviors of Al/ZnO NPs/Al optoelectronic synaptic devices with as-deposited, nitrogen-annealed (500 °C), and oxygen-annealed (500 °C) ZnO films, respectively, along with the RS I-V curves illustrating the optical set and electrical reset processes. First, a voltage sweep from 0 to 1.0 V is applied to the Al/ZnO NPs/Al device, followed by UV illumination at 0.7 V, inducing a transition from the high-resistance state (HRS) to the low-resistance state (LRS), referred to as the “optical set” process. After turning off the UV light, the applied voltage is gradually reduced from 1.0 V to 0 V, followed by sequential application of reverse voltages at −1.0, −2.0, −3.0, and −5.0 V before returning to 0 V. This reverse voltage application removes the LRS formed by the photoexcited carriers, restoring the device to HRS, a process known as an “electrical reset”. As shown in Figure 4a, the as-deposited Al/ZnO NPs/Al device exhibited a more than tenfold increase in current, rising from 0.84 nA to 9.23 nA when UV light was applied at 0.7 V under forward voltage bias. This significant increase in conductivity is attributed to the generation of photoexcited carriers in the ZnO NPs, leading to a sharp rise in current and a transition to LRS. Upon applying a reverse voltage, the current initially remains high due to the presence of trapped photoexcited carriers. At −1.0 V, when the reverse voltage is returned to 0 V, the operating current remains largely unchanged, indicating that photoexcited carriers do not dissipate at low reverse voltages, making electrical reset difficult. However, as the reverse voltage increases from −1.0 V to −5.0 V and returns to 0 V, the current decreases from 2.94 nA to 1.33 nA at −1.0 V, demonstrating that the trapped photoexcited carriers can be gradually released, restoring the device to HRS [35]. These results confirm that ZnO NPs exhibit a PPC phenomenon, where photoexcited carriers are retained due to trapping in deep-level states such as oxygen vacancies and Zn interstitials, or due to suppressed oxygen readsorption on the surface [36]. This trapping mechanism allows prolonged carrier retention, enabling not only resistance state storage [37] but also its erasure through electrical reset. Figure 4b,c illustrate the RS behaviors of devices annealed at 500 °C in O_2_ and N_2_ atmosphere, respectively. Devices annealed in both atmospheres exhibited a more pronounced transition from HRS to LRS compared to as-deposited devices, attributed to the optical set process induced by UV excitation. While the magnitude of the HRS to LRS transition during the optical set process remained nearly identical regardless of the annealing atmosphere, the electrical reset process revealed distinct differences. The 500 °C-O_2_ annealed device exhibited a significant increase in resistance at −1.0 V, from 3.04 × 10^6^ Ω to 7.19 × 10^6^ Ω, as the electrical reset voltage was increased from −1.0 V to −5.0 V. This indicates that oxygen-annealed devices can be effectively reset by applying a reverse voltage, restoring them to HRS. In contrast, the 500 °C-N_2_ annealed device, even with an increase in reverse voltage from −1.0 V to −5.0 V, the resistance change was minimal, increasing only slightly from 2.08 × 10^6^ Ω to 2.41 × 10^6^ Ω at −1.0 V. Figure 4d presents the resistance ratio (R_HRS_/R_LRS_) at −1.0 V. When the reset voltage was increased from −1 V to −5 V to evaluate the RS behavior, the as-deposited device exhibited a moderate increase in R_HRS_/R_LRS_ from 1 to 1.97, while the 500°C-O_2_ annealed device showed a more significant increase from 1 to 2.34, indicating effective reset functionality. In contrast, the 500 °C-N_2_ annealed device displayed minimal change, with R_HRS_/R_LRS_ increasing only slightly from 1 to 1.15, suggesting that reset formation is significantly suppressed in nitrogen-annealed devices. This further confirms that nitrogen annealing leads to strong carrier trapping in deep levels and reduced oxygen readsorption [31], preventing the transition back to HRS and sustaining the LRS even under high reset voltages. This suggests that nitrogen annealing enhances PPC properties, which contribute to prolonged carrier retention and suppressed reset functionality. Figure 4e–g present the RS I-V curves of as-deposited, 500 °C-O_2_ annealed, and 500 °C-N_2_ annealed devices, respectively, measured over 10 cycles with the optical set process (UV on at 0.7 V) and electrical reset process (−5.0 V). These results confirm that the optical set and electrical reset processes in optoelectronic synaptic devices, based on as-deposited and N_2_-/O_2_-annealed ZnO NPs, exhibit reliable and repeatable switching characteristics. 

To further analyze the switching stability, Figure 4h shows the HRS and LRS values at −1.0 V. The as-deposited and 500 °C-O_2_ annealed devices maintain a stable resistance state across repeated measurements. However, the 500 °C-N_2_ annealed device exhibits a small offset between HRS and LRS, with both resistance states gradually decreasing over repeated cycles. This behavior is attributed to the strong PPC effect in nitrogen-annealed devices, where trapped photogenerated carriers remain active, hindering complete electrical reset. As a result, the resistance continuously decreases with repeated optical sets and electrical reset cycles. For nitrogen-annealed devices, applying a sufficiently strong reset process is expected to increase the HRS/LRS offset, enabling prolonged retention of photogenerated carriers. This characteristic is particularly beneficial for long-term memory applications in optoelectronic synaptic devices.

### 3.5. Short-Term Synaptic Plasticity in Al/ZnO NPs/Al Optoelectronic Synaptic Devices and the Influence of Thermal Annealing on Paired-Pulse Facilitation 

**Figure 5:**
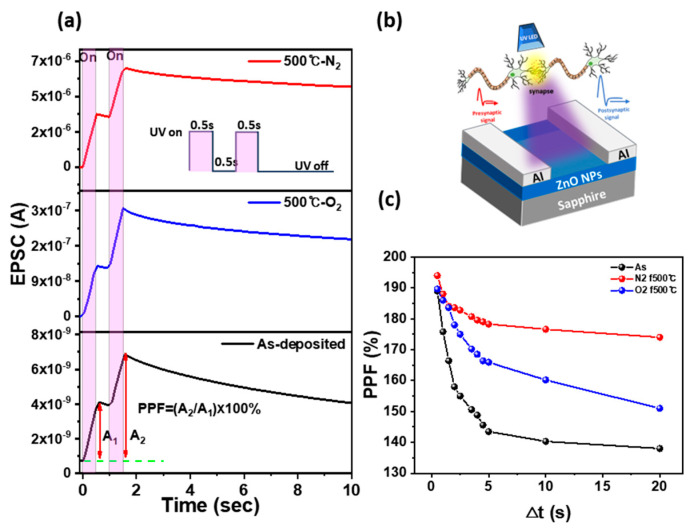
(**a**) EPSCs of ZnO NP-based optical synaptic devices with different annealing temperatures, were measured at 3.0 V using two consecutive light pulses with 0.5 s exposure and 0.5 s off intervals. (**b**) Schematic illustration of an Al/ZnO NPs/Al optical synaptic device and the transmission of presynaptic and postsynaptic signals at the synapse. (**c**) PPF as a function of different time intervals under a constant 0.5 s pulse exposure time for as-deposited, 500 °C-N_2_ annealed, and 500 °C-O_2_ annealed ZnO NP-based synaptic devices.

The short-term synaptic plasticity of Al/ZnO NPs/Al optoelectronic synaptic devices was evaluated by measuring changes in synaptic weight as a function of the time interval between two consecutive UV pulses with varying exposure durations [38], as shown in Figure 5a. Paired-pulse facilitation (PPF) was quantified as the ratio of the excitatory post-synaptic current (EPSC) elicited by the second stimulus (A_2_) to that elicited by the first stimulus (A_1_). A higher A_2_/A_1_ ratio indicates an increase in PPF, signifying enhanced short-term plasticity in synaptic connections. Figure 5b presents a schematic illustration of the Al/ZnO NPs/Al optoelectronic synaptic device fabricated on a sapphire substrate. The device emulates biological synaptic behavior, where UV light stimulation functions as a presynaptic signal, modulating the EPSC to mimic synaptic plasticity in neurons. The UV LED acts as an optical stimulus, inducing synaptic responses analogous to neurotransmission in a human synapse. Figure 5a presents the EPSC values over time for as-deposited, 500 °C-N_2_ annealed, and 500 °C-O_2_ annealed ZnO NP-based optoelectronic synaptic devices under UV illumination with an on/off interval of 0.5 s. The as-deposited device exhibits a PPF of 184%, accompanied by a rapid decline in the EPSC slope, indicating weak short-term retention. In contrast, the 500 °C-N_2_ annealed device demonstrates an enhanced PPF of 194%, suggesting improved synaptic plasticity due to increased carrier trapping and prolonged charge retention. Meanwhile, the 500 °C-O_2_ annealed device shows a PPF of 186%, similar to the as-deposited device, indicating that oxygen annealing has a minimal effect on short-term synaptic enhancement. In addition, the as-deposited devices exhibit a relatively low EPSC upon UV illumination and a rapid decline after UV off, whereas 500 °C-annealed devices show a higher EPSC and slower decay rate. This improvement is attributed to the enhanced crystallinity due to thermal annealing and the PPC effect induced by oxygen vacancies. Figure 5c presents the PPF values as a function of increasing UV exposure interval time (Δt) from 0.5 s to 20 s for as-deposited, 500 °C-N_2_ annealed, and 500 °C-O_2_ annealed devices. As the interval time increases to 20 s, the PPF of the as-deposited device decreases significantly from 184% to 138%, whereas the 500 °C-N_2_ annealed device exhibits a more gradual decline from 194% to 174%, and the 500 °C-O_2_ annealed device decreases from 184% to 151%. These results indicate that annealed ZnO NP-based optoelectronic synaptic devices exhibit less pronounced PPF degradation over time compared to as-deposited devices, demonstrating improved short-term synaptic plasticity and enhanced stability. Furthermore, compared to oxygen annealing, nitrogen annealing appears to increase oxygen vacancies, facilitating carrier trapping at deep levels while suppressing oxygen readsorption. This results in prolonged charge retention, leading to an increase in PPF, and further enhancing short-term plasticity.

### 3.6. Photostimulation-Dependent Synaptic Plasticity in ZnO NP-Based Optoelectronic Synaptic Devices and the Impact of Thermal Annealing on Memory Retention

**Figure 6:**
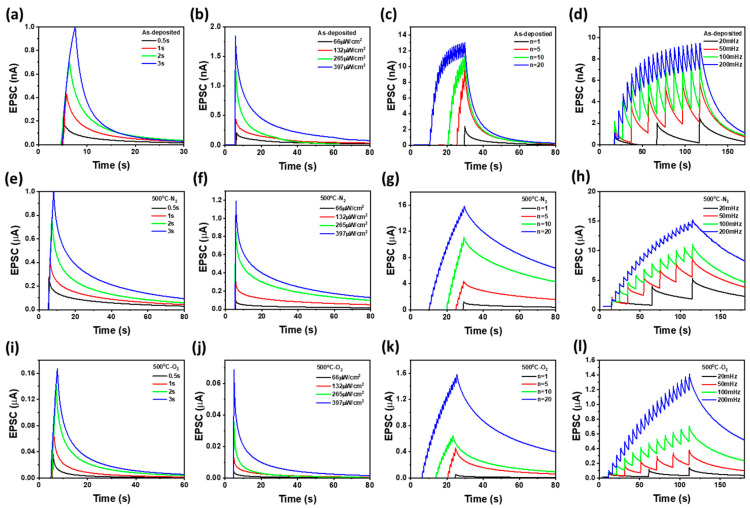
EPSCs of ZnO NP-based optical synaptic devices under different conditions for (**a**–**d**) as-deposited, (**e**–**h**) 500 °C-N_2_ annealed, and (**i**–**l**) 500 °C-O_2_ annealed devices. The measurements were performed by varying (**a**,**e**,**i**) exposure time, (**b**,**f**,**j**) light source power, (**c**,**g**,**k**) number of exposures, and (**d**,**h**,**l**) exposure frequency.

Figure 6a–d, e–h, and i–l present the time-dependent EPSCs of as-deposited, 500 °C-O_2_ annealed, and 500 °C-N_2_ annealed ZnO NP-based optoelectronic synaptic devices, respectively, in response to different photon stimuli-UV exposure time, excitation light power, frequency, and number of pulses. The optical potentiation and depression characteristics were analyzed by varying the 365 nm UV exposure time (0.5 to 3 s), light intensity (66 µW/cm^2^ to 397 µW/cm^2^ with a fixed exposure time of 1.0 s), number of pulses (1 to 20) with a pulse width of 0.5 s and 50% duty cycle, and frequency (20 mHz to 200 mHz) while maintaining a constant pulse width of 0.5 s. These measurements demonstrate that the photocurrent response in ZnO NPs thin films enables the transition of short-term memory into long-term memory, highlighting their potential for synaptic memory applications [6]. Compared to 500 °C-annealed devices, as-deposited devices exhibit lower EPSC values and faster EPSC decay, indicating their limited ability to convert short-term memory into long-term memory. In contrast, 500 °C-N_2_ annealed ZnO NP-based optoelectronic synaptic devices exhibit higher EPSC values and slower decay compared to 500 °C-O_2_ annealed devices, suggesting improved charge retention. This improvement is attributed to the increased photoexcited carrier generation and enhanced PPC due to nitrogen annealing, which suppresses deep-level trapping and reduces oxygen re-adsorption by creating oxygen vacancies. In contrast, in an oxygen atmosphere, the formation of oxygen complexes compensates for oxygen vacancies, reducing charge transport barriers and enhancing carrier mobility, thereby leading to a faster decrease in EPSC [13,14]. Additionally, in nitrogen-annealed devices, the higher density of photoexcited carriers suppresses oxygen adsorption after UV termination, slowing the decay of photogenerated current and resulting in a more sustained EPSC. Furthermore, the consistently high EPSC values observed with increasing photostimulation time, light intensity, frequency, and number of pulses, along with prolonged EPSC retention after photostimulation, indicate that ZnO NP-based optoelectronic synaptic devices exhibit strong charge retention, suggesting their potential for long-term memory applications.

### 3.7. Enhanced Learning and Memory Retention in ZnO NPs-Based Optoelectronic Synaptic Devices Through Thermal Annealing 

**Figure 7:**
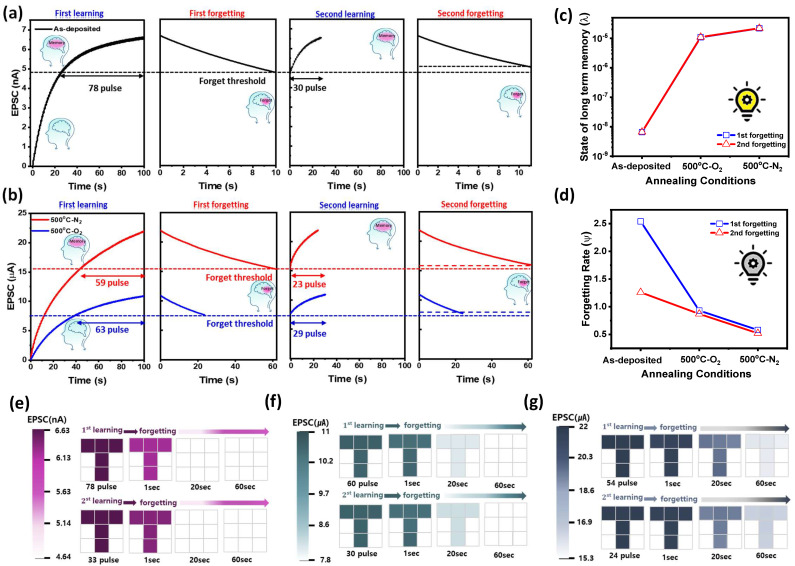
Measured learning and forgetting behaviors in response to optical stimuli for ZnO NP-based optical synaptic devices: (**a**) as-deposited and (**b**) 500 °C-O_2_ and 500 °C-N_2_ annealed devices. A pulsed optical stimulus (365 nm UV light with a pulse width of 0.5 s and a 50% duty cycle) was applied 100 times to induce the first learning phase, followed by two cycles of forgetting after turning off the UV light. (**c**) Learning degree and (**d**) forgetting rate are plotted as a function of thermal treatment conditions using Wickelgren’s power law. A 3 × 3 pixel array composed of nine Al/ZnO NPs/Al devices selected from three wafers containing (**e**) as-deposited, (**f**) 500 °C-O_2_ annealed, and (**g**) 500 °C-N_2_ annealed ZnO NP devices.

Figure 7a,b present the simulated learning and forgetting processes in as-deposited and 500 °C-N_2_/O_2_ annealed ZnO NPs-based optoelectronic synaptic devices, respectively. During the learning process, a 365 nm UV pulse train of 100 cycles (pulse width: 0.5 s, duty cycle: 50%) is applied to reach the maximum EPSC. In contrast, the forgetting process occurs naturally when the UV light is turned off, leading to a gradual decrease in synaptic weight and, consequently, a reduction in EPSC. A successful learning process is defined when the EPSC reaches at least 70% of its maximum value while forgetting is classified when the EPSC naturally decays below 70% during the forgetting process [39]. As shown in Figure 7a, the as-deposited ZnO NP-based optoelectronic synaptic device reached a maximum EPSC of 6.7 nA after 100 pulses. It required 78 pulses to increase the EPSC from 4.8 nA (70% of the maximum EPSC, the learning threshold) to 6.7 nA. After achieving the maximum EPSC, the forgetting process, characterized by natural depression upon turning off the UV light, resulted in a decay of 70% of the maximum EPSC within 10 s. During the secondary learning process, optical potentiation was applied at 70% (4.8 nA) of the maximum EPSC under the same pulse conditions as in the first learning process. Notably, the number of pulses required to reach the maximum EPSC decreased to 30, indicating an accelerated learning process with repeated training. Furthermore, in the secondary forgetting process, the time for the EPSC to decay to 70% of the maximum EPSC increased to 15 s, demonstrating a slower forgetting rate compared to the initial learning cycle. These results suggest that Al/ZnO NPs/Al optoelectronic synaptic devices exhibit improved learning efficiency and enhanced memory retention with repeated UV exposure, effectively mimicking biological synaptic plasticity. As shown in Figure 7b, the ZnO NP-based optoelectronic synaptic devices annealed at 500 °C in nitrogen and oxygen atmospheres exhibited a significant increase in EPSC, reaching 22 µA and 10 µA, respectively, after 100 training pulses (0.5 s pulse width and 50% duty cycle), compared to the as-deposited ZnO device, which showed an EPSC of 6.4 nA. Additionally, the number of pulses required to increase the EPSC from the learning threshold (70% of the maximum EPSC) to its peak value was significantly reduced for annealed devices, requiring 59 pulses for N_2_-annealed ZnO and 63 pulses for O_2_-annealed ZnO, compared to 78 pulses for the as-deposited device. During the forgetting process, the time required for the EPSC to decay to 70% of the maximum value was extended to 60 s for N_2_-annealed ZnO and 23 s for O_2_-annealed ZnO, compared to 10 s for the as-deposited device. In the second learning process, the number of UV pulses required to reach 100% EPSC was further reduced to 23 pulses for N_2_-annealed ZnO and 29 pulses for O_2_-annealed ZnO NPs-based optoelectronic synaptic devices, indicating an accelerated learning process with repeated training. Similarly, during the second forgetting process, the extinction time was significantly extended, reaching 90 s for N_2_-annealed ZnO and 35 s for O_2_-annealed ZnO NPs devices. Notably, during the first forgetting process, the EPSC of the N_2_-annealed ZnO NPs device remained above 70% even after 60 s of UV termination, which is approximately 4.5 times longer than the retention time (10 s) of the as-deposited ZnO NPs device. This further demonstrates that thermal annealing enhances long-term memory (LTM) properties, with the N_2_ annealing process particularly improving charge retention and memory stability. Moreover, Wickelgren’s power law has been employed to quantify the forgetting rate of Al/ZnO NPs/Al optoelectronic synaptic devices, a model widely used to describe biological forgetting. The equation is expressed as follows (Equation (1)) [40,41]:

I = λ × (1 + β × t)^Ψ^ (1)

where I represents the memory intensity, t is the decay time, λ denotes the degree of learning or the long-term memory state at t = 0, β is the scaling parameter, and Ψ represents the forgetting rate. Figure 7c,d illustrate the degree of long-term memory (LTM) and the forgetting rate of the device as a function of thermal treatment conditions. As shown in Figure 7c, the long-term memory state of the first- and second forgetting process in the as-deposited devices significantly increased from 6.59 × 10^−9^ to 1.08 × 10^−5^ and 2.18 × 10^−5^, respectively, following 500 °C-O_2_/N_2_ annealing. Additionally, Figure 7d shows that the forgetting rate during the natural depression process was significantly reduced in 500 °C-annealed devices compared to as-deposited devices. Notably, devices annealed in a nitrogen atmosphere exhibited a lower forgetting rate than those annealed in oxygen, in both the first and second forgetting processes. It is also observed that the forgetting rate decreased progressively with each subsequent forgetting cycle under all annealing conditions. This enhancement in long-term memory retention and reduction in the forgetting rate is attributed to the improved crystallinity of ZnO NPs with increasing annealing temperature, particularly in a nitrogen atmosphere. The enhanced crystallinity facilitates greater photoexcited carrier generation, thereby strengthening long-term memory retention. Furthermore, the lower forgetting rate in nitrogen-annealed devices is associated with the trapping of photoexcited carriers at deep levels and the suppression of oxygen readsorption, effectively prolonging the retention of stored information [42,43]. Figure 7e–g illustrate the layout of the in-sensing memory, consisting of a 3 × 3 pixel array composed of nine Al/ZnO NPs/Al devices selected from three wafers containing as-deposited, 500 °C-O_2_, and 500 °C-N_2_ annealed ZnO NP devices. Based on the results in Figure 7a,b, the EPSC values generated by the pulse input were used to encode the intensity of each pixel color, with brightness levels adjusted to represent the degree of learning. This encoding was further validated by the residual EPSC values after the excitation light was turned off following the first learning process, revealing a gradual color fade over time, indicative of the forgetting process. The as-deposited ZnO NP devices were initially encoded in very dark violet colors during the first learning process but faded to very light colors during the subsequent forgetting process, reaching the forgetting threshold EPSC within just 10 s. However, during the second learning and forgetting process, they were encoded in noticeably darker colors within the same 10 s period, indicating improved retention. Similarly, ZnO NP devices annealed in oxygen and nitrogen atmospheres were initially encoded in very light colors at their first forgetting thresholds of 20 s and 60 s, respectively. However, after the second learning process, the forgetting process resulted in darker color retention for the same duration, demonstrating enhanced memory retention with repeated learning. These results demonstrate that thermal treatment significantly improves LTM characteristics in ZnO NP-based optoelectronic synaptic devices. Furthermore, N_2_-annealing is more effective than O_2_-annealing in enhancing LTM due to increased PPC properties. 

## 4. Conclusions

This study demonstrates that thermal annealing at 500 °C significantly enhances the structural, optical, and optoelectronic properties of ZnO NP-based devices, with nitrogen annealing playing a crucial role in improving memory retention. AFM analysis reveals that annealing increases grain size while reducing surface roughness, with oxygen-annealed films exhibiting larger grains and smoother surfaces. UV-visible spectroscopy and PL measurements confirm a blue shift in the absorption edge and enhanced near-band-edge emission, particularly in nitrogen-annealed samples, due to increased oxygen vacancies and improved crystallinity. XPS analysis further supports this by showing higher concentrations of oxygen vacancies in nitrogen-annealed films, facilitating charge carrier trapping and enhancing PPC. Electrical and optoelectronic measurements indicate that nitrogen-annealed devices exhibit higher EPSC, slower decay, and extended retention compared to oxygen-annealed and as-deposited devices. I-V characteristics demonstrate that nitrogen annealing suppresses oxygen readsorption and enhances charge retention, leading to stronger PPC and more stable RS behavior. Furthermore, optical resistive switching RS characteristics confirm that nitrogen-annealed devices achieve a more pronounced LRS-to-HRS transition under reverse voltage application due to higher PPC and suppressed oxygen adsorption. Learning and forgetting simulations show that nitrogen-annealed devices require fewer pulses for learning and retain memory longer. In-sensing memory analysis using a 3 × 3 pixel array further illustrates improved memory retention through brightness encoding. These findings highlight ZnO NP-based optoelectronic synaptic devices as promising candidates for neuromorphic computing, with nitrogen annealing proving to be a key factor in optimizing their long-term memory performance through oxygen vacancy engineering.

## Data Availability

The original contributions presented in this study are included in the article. Further inquiries can be directed to the corresponding author.
